# Development of an Australian FASD Indigenous Framework: Aboriginal Healing-Informed and Strengths-Based Ways of Knowing, Being and Doing

**DOI:** 10.3390/ijerph20065215

**Published:** 2023-03-22

**Authors:** Nicole Hewlett, Lorian Hayes, Robyn Williams, Sharynne Hamilton, Lorelle Holland, Alana Gall, Michael Doyle, Sarah Goldsbury, Nirosha Boaden, Natasha Reid

**Affiliations:** 1 Child Health Research Centre, The University of Queensland, Brisbane, QLD 4072, Australia; 2 Curtin Medical School, Curtin University, Perth, WA 6000, Australia; 3 Faculty of Health, School of Nursing and Midwifery, University of Technology Sydney, Ultimo, NSW 2007, Australia; 4 School of Nursing, Midwifery and Social Work, The University of Queensland, Brisbane, QLD 4072, Australia; 5 National Centre for Naturopathic Medicine, Faculty of Health, Southern Cross University, Lismore, NSW 2480, Australia; 6 Faculty of Medicine and Health, University of New South Wales, Sydney, NSW 2052, Australia; 7 Māori/Indigenous Health Innovation, University of Otago Christchurch, Christchurch 8013, New Zealand; 8 Faculty of Social Work, The University of New South Wales, Sydney, NSW 2052, Australia

**Keywords:** fetal alcohol spectrum disorder, healing, wellbeing, strengths-based, Aboriginal, indigenous, cultural framework, assessment, Dadirri, yarning

## Abstract

Aboriginal culture intuitively embodies and interconnects the threads of life that are known to be intrinsic to human wellbeing: connection. Therefore, Aboriginal wisdom and practices are inherently strengths-based and healing-informed. Underpinned by an Indigenist research methodology, this article presents findings from a collaboration of Aboriginal and non-Aboriginal peoples to develop an Australian Fetal Alcohol Spectrum Disorder (FASD) Indigenous Framework during 2021 to 2023. The FASD Indigenous Framework unfolds the changes that non-Aboriginal clinicians and Aboriginal peoples each need to make in their respective ways of knowing, being and doing in order to facilitate access to healing-informed, strengths-based and culturally responsive FASD knowledge, assessment, diagnosis and support services among Aboriginal peoples. Drawing on the Aboriginal practices of yarning and Dadirri, written and oral knowledges were gathered. These knowledges were mapped against Aboriginal cultural responsiveness and wellbeing frameworks and collaboratively and iteratively reflected upon throughout. This article brings together Aboriginal wisdom (strengths-based, healing-informed approaches grounded in holistic and integrated support) and Western wisdom (biomedicine and therapeutic models) in relation to FASD. From a place of still awareness (Dadirri), both forms of wisdom were drawn upon to create Australia’s first FASD Indigenous Framework, a new practice in the assessment and diagnosis of FASD, which offers immense benefit to equity, justice, support and healing for Aboriginal families with a lived experience of FASD.

## 1. Introduction

For millennia, Aboriginal ways of knowing, being and doing have been fundamental to sustaining the oldest living culture in the world [1]. Aboriginal wisdom and practices are inherently strengths-based and healing-informed, as they intuitively embody and interconnect the threads of life that are known to be intrinsic to wellbeing [1,2,3]. The spirit of a healing-informed approach is the unwavering truth that within Aboriginal individuals, families and communities exist unique cultural values, beliefs, traditions, customs and practices that promote natural pathways of healing [2,4,5]. Aboriginal culture offers a foundation to heal the physical, emotional, mental and spiritual scars imposed by colonisation [5,6]. One of the most insidious scars is the extensive, complex and compounding effects of historical and contemporary trauma from which fetal alcohol spectrum disorder (FASD) was borne [5,7,8,9]. FASD is a diagnostic term used to describe the lifelong impacts on the brain and body that individuals can experience following prenatal exposure to alcohol [10]. The lifelong impacts on cognition, behaviour and learning experienced by those with FASD can manifest in academic, emotional and social difficulties, which often underpin challenges at home, school, work and in the community [11]. Given that FASD can present with or without physical abnormalities, these challenges can be profoundly exacerbated when FASD goes unrecognised [12,13]. It is important to note that not all people who are exposed to alcohol during pregnancy will have FASD. The range and severity of the impacts on the brain and body are as varied, as they are complex, with comorbidities being the “rule rather than the exception for people with FASD” [14] (p. 356). Every individual with FASD is unique, with many strengths, positive characteristics, talents and abilities that can contribute to their wellbeing when they have access to FASD diagnosis and appropriate supports [15].

### 1.1. Australian FASD Narratives as A Barrier to Accessing Knowledge, Diagnosis and Support

The negative and deficit narratives that exist in Australian society around prenatal alcohol exposure and FASD have generated blame on, and shame for, mothers of children with FASD [7,16]. Further, children who have a FASD diagnosis are often subjected to negative stigma due to unfair misconceptions about this condition and its impact on the individual [7]. For example, there is a belief that children with FASD will “become adults who could be dangerous in society” [7] (p. 34). In Australia, this fear has a silencing effect at the individual, family, community, organisational and policy levels, whereby it is socially unacceptable, and even controversial, to speak about alcohol use in pregnancy and/or FASD [13,16]. It can be uncomfortable asking anyone about alcohol use during pregnancy generally, however, when it comes to asking Aboriginal peoples about alcohol use during pregnancy, this “uncomfortable” feeling becomes paralysing, and many health professionals report avoiding the topic altogether [17]. On one hand, this stems from a lack of awareness, and therefore, confidence among health professionals to discuss the risk of alcohol use during pregnancy [17]. On the other hand, it comes from a place of health professionals’ not wanting to cause further harm or stigmatisation. In practice, both these hands translate into a systemic barrier to accessing accurate health knowledge, whereby health professionals do not provide correct advice about alcohol use in pregnancy, if at all. Further, organisations and policy makers often avoid associating the words “Aboriginal” with “FASD” [9]. In situations where discussions about prenatal alcohol exposure are not approached in a sensitive manner, Aboriginal peoples who may already be living with unprecedented levels of stigma, poverty, discrimination and racism can be triggered by health professionals [5]. Any discriminatory, judgemental and disrespectful language, approaches and attitudes that are used when discussing alcohol use or FASD can intensify Aboriginal peoples’ distress [5,7,8]. This serves to reinforce a negative experience of accessing health services and discourage health-seeking behaviour among Aboriginal peoples [5,7,8].

### 1.2. Aboriginal Healing-Informed and Strengths-Based Pathways

Healing-informed pathways are required in Australia to effectively address FASD and untangle the societal narratives that percolate, unquestioned, beneath the surface of what is “socially acceptable” [16]. Societal-level change requires a pathway where Aboriginal and Western wisdom can come together in a meaningful and equitable collaboration and knowledge exchange [18]. Healing for Aboriginal peoples is underpinned by holistic ways of knowing, being and doing, which emphasise the interconnectedness between the mental, physical, emotional, social and spiritual aspects of a person’s life, as well as their reciprocal relationships with family, community and Country (i.e., land and waters) [1,2,6,19]. In Aboriginal communities, healing is based on wellness rather than illness. Accordingly, good health and wellness/wellbeing means more than the absence of diseases, highlighting the strengths-based world-view that is inherent in Aboriginal culture [6]. It stands to reason that any healing-informed strategies must be underpinned by the aim to restore balance and harmony in all parts of life considered to be important to Aboriginal wellbeing, according to Aboriginal peoples [2,19].

Among Indigenous peoples globally, strengths-based approaches that centre on meeting cultural needs, which are intrinsically healing-informed, have been found to decrease stigma, strengthen relationships, enhance cultural identity and connection, promote hope, measure cognitive abilities more accurately, lessen stress and feelings of hopelessness and support healthier coping for families [5,13,15,20,21]. Despite this, there remains a lack of strengths-based research and approaches to FASD, which has been found to perpetuate stigma, exacerbate mistrust of the healthcare system [4] and significantly undermine Aboriginal peoples’ access to health services [3,7,15,22]

### 1.3. Australian Guide to the Diagnosis of FASD

In 2020, the Australian Government funded a review of the 2016 Australian Guide to the Diagnosis of FASD (hereafter referred to as the 2016 Guide) to ensure it reflects current knowledge and national and international best practices [23,24]. The aim of the 2016 Guide was to build the capacity of healthcare practitioners to consider a diagnosis of FASD, make a diagnosis and manage/refer an individual or family living with FASD [23,24]. All individuals with FASD can benefit from a multidisciplinary assessment process involving medical professionals, psychologists, occupational therapists, nurses, social workers and speech therapists to accurately assess, diagnose and support people across the lifespan [23]. For Aboriginal peoples with FASD, there are unique and important historical and cultural considerations that must be taken into account. Firstly, it is important to consider how different experiences of history flows through to current attitudes that see Aboriginal peoples as “other” or the object of pity and/or shame [25]. The second consideration is for the entrenched racism that goes largely unacknowledged within institutional structures and processes. Institutionalised racism critically shapes Aboriginal relationships with mainstream service providers and can cultivate patterns of ill-health and impairment for Aboriginal peoples [25]. While the aims of the 2016 Guide supported healthcare practitioners to apply Western assessment and diagnostic approaches, Aboriginal voices were lacking in recommended practices, risking the perpetuation of institutionalised racism in established clinical practices around FASD diagnosis [23,24].

The aim of the current article is to describe the process that was undertaken to develop an Australian Fetal Alcohol Spectrum Disorder Indigenous Framework. This solutions-focused, action-oriented resource was developed to unpack what non-Aboriginal clinicians and Aboriginal peoples need to know, be and do in order to increase access to trauma-aware and healing-informed culturally responsive assessments and FASD diagnosis among Aboriginal peoples. This article argues that by considering the concepts described in the FASD Indigenous Framework and employing an Aboriginal healing-informed approach, stigmatising and racialised health practices will be reduced, and positive outcomes for Aboriginal peoples will be realised. Although efforts were made, no Torres Strait Islander person was available to contribute to the development of the FASD Indigenous Framework. Thus, with deepest respect and in the spirit of honesty and transparency, this study and the current FASD Indigenous Framework speaks only from Aboriginal perspectives in Australia.

## 2. Materials and Methods

### 2.1. Knowledge Holders

In the spirit of Aboriginal ways of knowing, being and doing, the current article shifts away from terminology such as “participants” to “knowledge holders” to respect the value, voice and sovereignty of knowledge holders’ stories and experiences [26]. Indigenist methodology challenges the Western narrative around what constitutes “adequate evidence”. Indeed, Aboriginal knowledge holders are known to hold many different roles in their communities, which are highly complex and interrelated. Aboriginal knowledge holders embody holism, in that each is a singular connected being made up of interconnected woven parts, representing the many roles and responsibilities that shape their cultural, physical, academic and spiritual knowledge. For example, an Aboriginal knowledge holder who contributed to the development of the FASD Indigenous Framework identified as an Aboriginal woman, mother, aunty, friend, support worker, daughter, sister, researcher, educator, cousin, kinship carer, health worker, healer, advocate and translator. These varied, reciprocal and interrelated roles become even more profound when an Aboriginal person becomes an Elder, proving that such knowledge is immeasurable. With deep respect and honour to the Aboriginal knowledge holders who embody holism and represent many dynamic and interconnected roles, this article challenges Western research methods that would seek to reduce such layered wisdom to a single participant. Of the authors, the knowledge holders who participated in the individual two-way yarns include LHa, RW, SH and SG. The authors who participated in the collaborative yarns include LHa, RW, SH, LHo, MD, SG, NB and NR.

### 2.2. The Authors

NH is a proud Palawa woman from the south-east coast of lutruwita (Tasmania) and pays her respects to the original custodians of Country on which this article was written, the Turrbul and Yuggera peoples of Meeanjin (Brisbane). NH also acknowledges and extends deep respect to her ancestral spirits, which have guided and held her to this very moment and are thoughtfully reflected throughout these pages. Although NH comes from a family impacted by entrenched cycles of intergenerational trauma on both sides, she was also gifted a mother who, without education, resources or support, never passed on so much as a whisper of the violence, disdain or abuse that was so severely inflicted upon her. To be raised by such a loving, gentle, but powerful woman gave NH the open heart to connect and surrender to *Muyini* (Great Spirit) and the ancestral wisdom that radiates from this Dreaming. It also shaped her way of knowing and being, to understand how the strength of culture, country and kinship serves to fortify and sustain all threads in the tapestry of her wellbeing. NH’s mother also instilled a deep knowing that healing intergenerational trauma cycles is possible and an even deeper passion for the “doing”, that is, to make this healing accessible to all Aboriginal peoples.

The knowledge held on these pages was informed by the unwavering generosity and ancient wisdom of Aunty/Dr. Lorian Hayes (LHa). LHa is a 7th generation Iningai/Bidjera woman from central western Queensland. Her bush name, *Murrindji,* means “holder of language, lore and knowledge” as it relates to health. Language, in this context, refers to the knowledge and lore held around FASD and translated to diverse communities. When representing *Murrindji*, an unfolding occurs of the many cultural dimensions of what it means to gift knowledge and how such a gift instils a vibration of health that resonates with those present. There is not a single area of the Aboriginal FASD space that has not been touched by the ripples of *Murrindji* in the past 51 years. 

RW is a Noongar woman and research advocate from the south-west of Western Australia, with kinship ties to the Kimberley region of WA. As a young woman, Williams lived and worked in an Aboriginal community and has documented this experience in her doctoral study [7]. Aboriginal communities have practised culture as intervention since time immemorial. Her PhD on FASD in Noongar country was completed in 2018.

SH is a Ngunnawal woman whose cultural connections are to Canberra and Yass. SH is an academic and child health researcher whose work focuses on FASD, cultural understandings of diagnosis, health and illness and equitable access to culturally and neurodevelopmentally relevant resources and support for families involved with the child protection and youth justice systems.

LHo is an Aboriginal woman with spiritual and cultural connections to Mandandanji Country (Roma Queensland). LHo is a registered nurse and academic whose research aims to prevent punitive punishment and the incarceration of Aboriginal and Torres Strait Islander children with complex needs inclusive of FASD.

MD is a Bardi Man. The Bardi people are from the West Kimberley region of Western Australia. MD is post-doctoral researcher with a focus on the criminal justice system.

AG is a Pakana woman from the north-east coast of lutruwita (Tasmania) and her spirit name is *kaparunina* (Tasmanian tiger). AG has over 11 years’ experience in global Indigenous health and wellbeing research, with a focus on Aboriginal traditional medicines and healing practices. AG has lived experience of FASD, growing up in a loving family that intuitively employed healing-informed strategies to support the neurodiverse children in their care.

SG is a Māori woman of Ngāti Porou and Te-Aitanga-a-Hauiti whakapapa (descent). SG is a clinical psychologist and neuropsychologist in Aotearoa New Zealand, where her clinical work and research focuses on FASD assessment with Māori children and adolescents. 

NB is a Sri Lankan–Sinhalese Australian woman, with over 10 years’ experience as both a mental health social worker and intellectual disability academic, specialising in FASD. NB has spent the last two years supporting culturally responsive access to child and adolescent mental health and neurodevelopmental assessments and FASD diagnoses in remote Aboriginal communities across the Northern Territory.

NR is a Caucasian woman from Western Queensland. NR’s advocacy for decolonised health practices and equity-focused accessibility to FASD services and supports has made her a well-respected friend, ally and advocate among many Aboriginal peoples, but especially the authors. The authors recognise that the opportunity for this research would not exist if it were not for NR’s dedication to making it so. NR is a researcher and clinical psychologist with experience in assessment, diagnosis and support of individuals with FASD and their families.

### 2.3. Methodology

The current FASD Indigenous Framework development process intuitively embodies Aboriginal ways of knowing, being and doing (i.e., an Indigenist research approach) [27], which underpins the aim of the research. In line with an Indigenist research approach, principles of resistance, Aboriginal leadership integrity and privileging Aboriginal voices were integrated while using and adapting Western methods [28]. Information was gathered, analysed and shared by an Aboriginal person (NH), under the mentorship of an Aboriginal Elder and senior researcher in FASD (LHa), an Aboriginal researcher in FASD (RW) and a non-Aboriginal FASD clinician and researcher (NR). All the work was supported and informed by a Cultural Advisory Group established to inform and guide the development of the Australian Guidelines for Assessment and Diagnosis of FASD. The current research centred on Aboriginal healing-informed practice of yarning (two-way communication) and Dadirri (deep listening) to enrich and develop an Australian FASD Indigenous Framework [29].

At the heart of the FASD Indigenous Framework is the ability for Aboriginal and non-Aboriginal peoples to come together and have a two-way, equal exchange with respect and a genuine openness to deep listening and learning. This calls upon two ancient, yet timeless, Aboriginal practices of Dadirri [29,30,31] and yarning [5,7,8,20,31,32,33]. Dadirri is a gift shared by Aunty/Dr. Miriam Rose Ungunmerr-Baumann, an Aboriginal Elder of Daly River (Northern Territory), who explains that Dadirri is an inner, deep listening and a quiet, still awareness that creates space for profoundly respectful and genuinely reciprocal relationships [30]. Dadirri serves as a healing-informed practice as it counters the effects of colonisation through a strengths-based truth-telling, which focuses on the empowerment, self-determination and resilience of Aboriginal communities [30]. By undertaking a journey of learning from listening and providing a purposeful plan to act, informed by wisdom and embraced by the responsibility that comes with knowledge, a richer understanding and knowledge grounded in truth and respect can flourish [30]. Critically, in order for truth and respect to go both ways, so must listening and learning; Aboriginal and Western knowledge must come together without one ruling the other one [18,29].

Yarning is a conversational exchange that draws on the deep listening and the quiet awareness of Dadirri, to facilitate a two-way process that can lead to the creation of new knowledge and understanding [29,30,32,33,34]. Each person in the yarn is equal and treated as respected knowledge holders in their own right [26]. As is Aboriginal way, the sharing of knowledge is solutions-focused, holistic and reciprocal, while layers of meaningful reflection, trust and connection are being built [17,26,27,28,29,30,31]. Aboriginal and non-Aboriginal peoples benefit from the spiritual, cultural, social and emotional wellbeing that yarning personifiesand, as such, yarning can be seen as a way of facilitating genuine healing [35]. Extending on the yarn is the concept of “collaborative yarning methodology”, a culturally responsive approach to data collection and analysis that is underpinned by the principle of self-determination [36]. Both individual and collaborative yarns were fundamental to developing cultural knowledge, which informed the FASD Indigenous Framework.

The principles of yarning and Dadirri set the tone for respectful and collaborative approaches to data collection, interpretation, analysis and conceptualisation of findings. To enable realistic, grassroots knowledge translation and clinical practice implementation, Indigenous Allied Health Australia’s (IAHA) Culturally Responsive Framework was also employed [37]. The IAHA Framework is a high-quality, action-oriented Aboriginal and Torres Strait Islander approach to cultural safety [37]. It is intrinsically transformative and driven by three principles: knowing (knowledge—what do we need to *know* to be culturally responsive); being (self-knowledge and behaviour—what do we need to *be* to be culturally responsive); doing (action—what do we need to *do* to be culturally responsive) [37]. Infusing the IAHA Framework throughout this research has embedded another layer of healing to the assessment and diagnostic process by finding practical strategies to create culturally safe spaces. By establishing trusting and two-way engagement with Aboriginal peoples, healing-informed pathways to equity and justice may be realised. Acknowledgement of the complexity of intergenerational trauma related to harmful colonial practices and the social and cultural determinants of health and wellbeing is central to trust building [22,38].

The IAHA Culturally Responsive Framework was also adapted and applied to understand what Aboriginal communities need to know, be and do to understand and access assessment and diagnosis of FASD. Drawing on a strengths-based, healing-informed approach to ensure that all the evidence was interpreted through an Aboriginal wellbeing lens, the Fabric of Aboriginal and Torres Strait Islander Wellbeing model was integrated into our approach [2]. The Fabric of Wellbeing model highlights eight dimensions or “threads” of life, identified by Aboriginal peoples as important to their wellbeing: belonging and connection, holistic health, purpose and control, dignity and respect and basic needs that are interwoven through one’s family, community and culture [2]. The model emphasises that an Aboriginal persons wellbeing is contingent on the strength of each individual thread of life, as well as the strength of the interconnections between these threads [2].

In the spirit of genuine two-way knowledge sharing, the development process also incorporated and adapted Western conceptual framework analysis [39]. Conceptual framework analysis is an iterative process that resonates with the cyclical and holistic approach of collaborative yarning [36]. It involves eight phases, however, in the current research, the fourth phase (i.e., deconstructing the concepts) was removed as it did not conceptually align with an Indigenist research approach. The following sections describes each phase and the journey undertaken to embed Aboriginal ways of knowing, being and doing throughout.

#### 2.3.1. Phase One: Mapping the Selected Data Sources

Three key data sources were identified for the current research: (1) group collaborative yarns, (2) a comprehensive literature review and (3) individual yarns. These three data sources form what this article categorises as a review of all the available knowledge, oral and written, referred to hereafter as the “knowledge review”. Collaborative yarns were held with the Cultural Advisory Group throughout all phases of the development process. The Advisory Group included nine Aboriginal members (eight FASD researchers and the CEO of First Nations Disability Network) and three non-Aboriginal members (a NACCHO representative, FASD clinician/researcher and FASD social worker/researcher).

A comprehensive scan of the multidisciplinary literature was conducted. The literature review focused on the assessment and diagnosis of FASD in Indigenous communities, nationally and internationally. The literature that focused only on aspects related to FASD (e.g., stigma) or were published by solely non-Aboriginal researchers were excluded. Accordingly, the literature whereby an Aboriginal person was the first author was prioritised and privileged. Members of the Cultural Advisory Group were also invited to submit any peer reviewed and/or grey literature they considered to be relevant to the review process. Based on the literature review, it was decided that individual yarns would be helpful to inform specific areas of the FASD Indigenous Framework development process. NH undertook the literature review, chaired the collaborative yarning sessions and facilitated the individual yarns.

#### 2.3.2. Phase Two: Extensive Reading and Categorising of the Selected Data

The identified literature and knowledge collected from the collaborative yarning process was read and categorised using the knowing, being and doing principles of the IAHA Culturally Responsive Framework and the dimensions of the Fabric of Aboriginal and Torres Strait Islander Wellbeing model, as per Table 1 [2]. Key findings from the literature review and individual yarns were mapped according to whether they built on the wellbeing domains of “Belonging and Connection”, “Holistic Health”, “Purpose and Control”, “Dignity and Respect” and “Basic Needs” among Aboriginal peoples with FASD. These domains are underpinned the “Knowing” and “Being” components of the Culturally Responsive Framework. Any practical actions that emerged from the selected data around the assessment and diagnosis of FASD were recorded under “Doing”. NH undertook this mapping process and was supported throughout with NR and RW.

#### 2.3.3. Phase Three: Identifying and Naming Concepts

A cyclical process was used during this phase, which included NH reading the literature, reflecting and sitting in stillness with the findings (i.e., practising Dadirri), holding one or more individual yarns, practicing Dadirri with what arose from the yarns and then re-reading the literature with a shifted perspective. The initial concepts that emerged from this process were tentatively named by NH. NH presented the draft concepts to the Cultural Advisory Group on 24 May 2022 to collaboratively yarn, reflect, refine and reform. Insights shared during this collaborative yarn highlighted gaps that had not been addressed by the initial concepts. Specifically, what do Aboriginal communities need to know, be and do to access assessments and diagnosis of FASD and how are Aboriginal cultural needs considered during a Western assessment and diagnosis of FASD. The outcomes of this collaborative yarn led to further individual yarns with Aboriginal knowledge holders, as well as areview of additional literature authored by Aboriginal peoples.

#### 2.3.4. Phase Four: Integrating the Concepts

Following the cyclical process used in phase three, phase four involved grouping similar concepts together and organising them into two groups (1) concepts that facilitated the knowing, being and doing of clinicians to deliver culturally responsive FASD knowledge, services and support and (2) concepts that facilitated the knowing, being and doing of Aboriginal communities to access culturally responsive FASD knowledge, services and support. This phase highlighted the critical need for the guidelines to reflect the visual and story-telling ways in which Aboriginal peoples understand and communicate knowledge. During this phase, the concepts were visually captured using Aboriginal designs which were informed by stories of strength in Aboriginal and Torres Strait Islander communities.

#### 2.3.5. Phase Five: Synthesis, Resynthesis and Making It All Make Sense

Phase five involved an extension of the ongoing and collaborative cyclical process used in phases three and four. Over a period of six months, NH’s insights, which were cultivated by the practice of Dadirri, were further supported and shaped by informal yarns with mentors (LHa, RW and NR). LHa and RW provided ongoing cultural support and wisdom to NH, which served two purposes: (1) to support, translate and evolve NH’s insights, ensuring their relevancy and realistic applicability to diverse Aboriginal communities at a grass roots level, and (2) to keep NH accountable to Aboriginal knowledge integrity and the fundamental Aboriginal values of respect and reciprocity. Similarly, NR provided significant clinical and research support to NH to ensure that the concepts could genuinely translate to a Western clinical service for diagnosing FASD. NR also supported NH’s understanding around the challenges clinicians face when they are diagnosing FASD. This facilitated NH’s ability to synthesise and resynthesise concepts that could be realistically used by the FASD clinical workforce, and hopefully, increase the confidence and uptake of the concepts.

#### 2.3.6. Phase Six: Validating the Conceptual Framework

The revised draft of the FASD Indigenous Framework, including the visual design, was presented to the Cultural Advisory Group on 1 September 2022. In line with Aboriginal protocols, consent from all Aboriginal members of the Cultural Advisory Group was required before NH could present to any other groups. Members provided feedback, support and approval for the knowledge contained in the FASD Indigenous Framework to be shared with non-Aboriginal Advisory Groups on the Australian Guidelines review project. The draft FASD Indigenous Framework was presented to the overarching Guideline Steering Committee on 8 September 2022 and to the remaining five Advisory Groups comprised of non-Aboriginal clinicians, researchers and people with lived experience of FASD (i.e., adults with FASD and parents/caregivers of individuals with FASD) on 15 September 2022. The final presentation was recorded and circulated to 114 members of the Australian Guidelines review project to capture as much multi-disciplinary and widely diverse feedback as possible. Feedback was recorded verbally and minuted during the meetings. Feedback was also received in writing, out of session, for those that could not attend meetings. All feedback was incorporated into the now dynamic FASD Indigenous Framework.

## 3. Results

### 3.1. Literature Review

The review of the literature identified sixteen peer reviewed journal articles (ten Australian articles [20,22,40,41,42,43,44,45,46,47], and seven international articles [9,15,21,41,48,49,50]), one Australian perspectives commentary [51] and four PhD theses (three Australian theses [5,7,8] and one international thesis [52]) relating to FASD and Indigenous peoples. No published or grey literature was found that focused on addressing FASD in the Torres Strait Islander population. The identified literature predominately contained information and recommendations for non-Aboriginal clinicians providing culturally responsive FASD information, services and support to Aboriginal peoples. There was very little information and advice for Aboriginal communities around accessing FASD information, services and support.

### 3.2. Collaborative Group and Two-Way Individual Yarns

Throughout the research process, eight collaborative yarns over a two-year period were held with the Cultural Advisory Group. These group yarns, alongside the individual two-way yarns, worked towards creating balance in the mix of Aboriginal and Western knowledges. Seven two-way yarns were held with knowledge holders (three Aboriginal FASD researchers, one Māori FASD clinician and two non-Aboriginal clinicians working in Aboriginal settings). While the intention was to hold the yarns to populate the FASD Indigenous Framework with Aboriginal knowledge, the yarns enriched the entire FASD Indigenous Framework withnew knowledge that was not in the available literature. Yarns with Aboriginal knowledge holders revealed what Aboriginal communities needed to know, be and do to access FASD knowledge, services and support. Non-Aboriginal knowledge holders who specialised in assessing, diagnosing or supporting Aboriginal children with FASD revealed practical insights and detailed “how” to apply culturally responsive, healing-informed and trauma-aware services.

### 3.3. Conceptual Model

**Figure 1:**
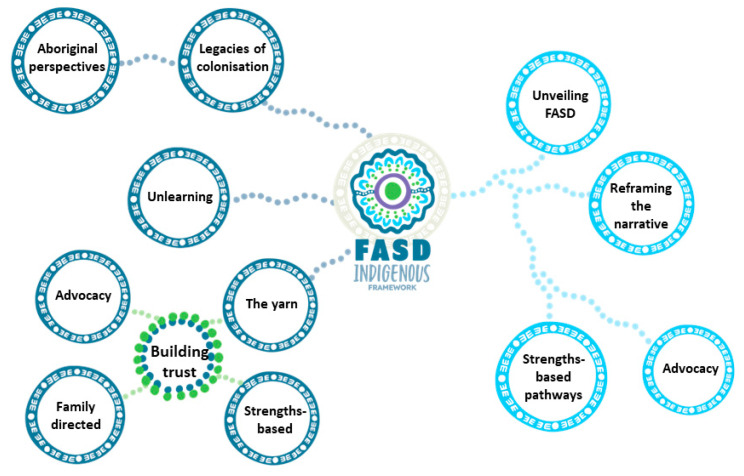
The current Australian FASD Indigenous Framework summarises the shifts non-Aboriginal clinicians and Aboriginal peoples need to make in their respective knowing, being and doing to facilitate access to FASD knowledge, services and support among Aboriginal peoples. The dark blue circles represent the shifts that non-Aboriginal clinicians need to make in their knowing, being and doing to deliver culturally responsive, healing-informed and trauma-aware FASD knowledge, services and support to Aboriginal peoples. The light blue circles represent the shifts that Aboriginal communities need to make in their knowing, being and doing to access FASD knowledge, services and support. The FASD Indigenous Framework visuals were designed by Worimi communication specialist Isaac Simons and non-Aboriginal graphic artist Daniel Richards. This community-informed design embodies the seamless flow of relationships and knowledge in Aboriginal and Torres Strait Islander communities and honours the strength of layered reciprocity and support that exists to nurture new life. The colours reflect the healing qualities of water and the vibrant and flourishing colours of fresh vegetation. The design captures the continuity of culture and encompasses the whole support process to highlight that everything is supported through connections with culture.

Figure 1 summarises the overall findings of what non-Aboriginal and Aboriginal peoples need to know, be and do to increase Aboriginal peoples’ access to culturally responsive FASD information, services and support. Overarching descriptions are provided below, with concepts included from the framework in bold. Supplementary Figure S1 presents the entire visual and includes all of the concepts that make up the Australian FASD Indigenous Framework As mentioned above, throughout the description of the framework, the term “knowledge review” refers to all the knowledge, written and oral, that was gathered, read and mapped against the areas in Table 1. The results briefly highlight findings that support the concepts of the FASD Indigenous Framework, and the discussion unpacks and articulates the key concepts and the vision in Figure 1 in greater detail.

### 3.4. What Clinicians Need to Know, Be and Do

#### 3.4.1. What Clinicians Need to Know

- Legacies of Colonisation

The knowledge review consistently found that to genuinely connect in a truly respectful way with Aboriginal peoples, it is critical that this connection stems from a place of truth-telling. This **truth-telling** is contingent on non-Aboriginal peoples having a critical knowledge of history and the profound legacies of **trauma, shame and guilt** that accompanied the weapons used to try and eliminate Aboriginal culture as part of colonisation [5,7,17,25,42,43,44,45,46,47]. Only then can there be a genuine understanding of the circumstances that lead to **developmental vulnerability** [46] among Aboriginal peoples, as well as the **deep medical mistrust** [5,7,8,9] held as a result of validated negative experiences with the Western system. Knowledge holder and Ngunnawal academic, Dr. Sharynne Hamilton, shared a critical point about detained children in a two-way yarn:

*“The impairments that these kids had were not necessarily solely related to FASD. You know, they have backgrounds of significant trauma and that might also be a significant contributing factor for why they might be impulsive, or why they can’t sit still or focus. You can’t diagnose FASD and wrap it all up in a nice shiny package with a ribbon on top. Untreated trauma is a significant issue that has major implications for a child’s neurodevelopment”.*

Knowing the legacies of colonisation in the Australian context was also found to highlight the critical importance of applying **strengths-based approaches as a healing-informed practice** [5,7,15,21,41,50]. When yarning with Indigenous (Aboriginal and Māori) knowledge holders and non-Aboriginal allies in the FASD space, the legacies of colonisation were an implied and shared understanding. Thus, legacies of colonisation were not specifically raised in the individual or collaborative yarns. However, the reviewed Australian literature [5,7,8,20,41,42,43,44,45,46,51] universally acknowledged the importance of understanding the impacts of colonisation and the connection between history and the current circumstances of Aboriginal families and communities. In her thesis, Dr. Sharynne Hamilton summarised that: “There is a need for Australia as a nation to confront its colonial history, decolonise justice and address systemic racism, and recognise the potential benefit of embracing Indigenous knowledge systems…” [5] (p.138). This finding is not unique to Australia, as a Māori knowledge holder, clinician and academic Sarah Goldsbury shared in a two-way yarn:

*“* *If we are to work effectively and appropriately with Māori, we need to understand the full context of Aotearoa’s history. We need to be aware of the systemic and institutional racism around us and within us. And what the impacts of this are for our clients and for our own roles, values, and beliefs as clinicians”.*

- Aboriginal perspectives

The second knowing pertained to non-Aboriginal clinicians considering Aboriginal perspectives and, therefore, culture. Understanding **Aboriginal worldviews** and definitions of **wellbeing,** including the critical importance of **respect and reciprocity**, **Country** and **family/kinship** [5,7,8,20,40,46,51], can potentially serve three purposes in the assessment and diagnosis of FASD. Firstly, it contributes to understanding the significant protective role that culture can play in achieving positive outcomes for Aboriginal peoples living with FASD [9,20,40,46]. Secondly, increasing the amount of knowledge and understanding of Aboriginal perspectives can support non-Aboriginal clinicians’ respect for, and pride in, the oldest living culture(s) in the world. Thirdly, this can, in turn, serve to reduce bias and cultivate a respectful connection with Aboriginal people and families [41,46,48,49]. It is essential to know that while Aboriginal peoples may have shared understandings and perspectives of the world, the **diverse identities** of Aboriginal peoples mean that each Aboriginal person varies in how they interpret, define and experience the threads of life that make up their wellbeing [5,7,8]. This highlights the importance of deeply listening to each Aboriginal person and family as a way of demonstrating genuine respect for and understanding of diverse Aboriginal culture while also acknowledging that Aboriginal peoples are experts in their own lives [45]. As knowledge holder and Noongar academic Dr. Robyn Williams highlighted in a two-way yarn:

*“On this journey, Aboriginal people need strong allies who become informed, and uphold Aboriginal worldviews and frameworks in the translation into clinical approaches and practice”.*

#### 3.4.2. What Clinicians Need to Be

- Unlearning

A key concept identified was the need to decolonise the automatic practices in mainstream settings that do not consider culture or strengths [5,7,8,15,41,46,50]. To deliver culturally responsive assessments, much of what needs to be developed in self-knowledge and behaviour (i.e., who non-Aboriginal clinicians need “to be”) largely focuses on an inward and often uncomfortable journey of decolonising and “unlearning” certain Western practices [7,41]. This unlearning pertains to the Western practices that have generated from untruthful narratives of colonisation and have given rise to the **bias, stereotypes and racism** that may consciously and unconsciously hide in the **power and privilege** of Western services and **assessment approaches** [5,7,8,41]. As Paradies has previously highlighted, “it is largely through societal systems of racism that colonial structures maintain material and symbolic (e.g., political) privilege” [53] (p.84). For example, assessments that are conducted as an interview with numerous questions typically assume a certain level of Western literacy and comprehension [42]. As knowledge holder and Ngunnawal academic Dr. Sharynne Hamilton emphasised in a two-way yarn:

*“Forget the questions because you will never get the answers or an honest account of what’s going on—these kids will tell you what they think you want to hear. Talk to them like you are talking to your own kids”.*

Further, Western assessment tools that have not been validated on Aboriginal populations may be inherently bias and exaggerate impairments, as Dr. Sharynne Hamilton explained:

*“We should assess kids by how well they do the things they are used to. So, thinking about assessing fine motor skills using handwriting, or with a pencil and paper. In some cases, kids that were being assessed came from very, very remote communities, where using a pencil and paper would have been very foreign to them. If their fine motor skills were assessed by, say, tying a fishing line, it could have been a very different outcome”.*

The knowledge review also highlighted that it is essential to unlearn one-way ”expert” knowledge sharing and move towards a two-way approach in clinical settings [45]. Part of this involves clinicians overcoming their **fear of making mistakes** that is often manifested by the traditional Western **clinician versus client relationship dynamic** [45]. As Māori knowledge holder Sarah Goldsbury shared:

*“Non-Indigenous clinicians must do much more than simply learn about the Indigenous cultures that they work with. It is essential they look within and identify their own cognitive biases from their life experiences and their clinical frameworks, and work to consistently unlearn and retrain responses stemming from these”.*

#### 3.4.3. What Clinicians Need to Do

The review found that building trust was central to delivering culturally responsive, healing-informed and trauma-aware assessments and diagnosis of FASD to Aboriginal peoples. All the practices that clinicians need to “do” were valued according to how effective each practice was at **building trust** with Aboriginal peoples. The review proposes that the following practices accumulatively build trust, that is, with each practice that is employed, another layer of connection and trust is built with Aboriginal peoples.

- The yarn

One of the most consistent findings across the literature and yarns was the necessity for non-Aboriginal peoples to be able to have a two-way, equal exchange with respect and a genuine openness to deep listening and learning [5,20,42,47]. Yarning [5,20,42,47] and **Dadirri** [5,42,47] are ancient, yet modern, ways of sharing Aboriginal knowledge that offer the opportunity of healing and two-way learning for those involved in the process. In this way, yarning is a practice of **healing-informed communication**. At the heart of yarning and Dadirri is **storytelling** and the ability for non-Aboriginal peoples to be vulnerable and share their stories to build two-way respect, connection and understanding so that trust can be cultivated [5,42]. Non-Aboriginal clinician Jade Houghton explained how she uses her story to build trust and connection with Aboriginal children undertaking an assessment of FASD:

*“So I’ll say “oh I’m from [hometown] and I’ve got a little sister and a big brother, I don’t live with mum and dad anymore because I’m old”. They get to know you and they think “okay she is sharing so that makes me feel more comfortable to share”. They know something about me and the kids begin to find the similarities and points of connection—they can relate to you”.*

For this trust to be built, it often requires non-Aboriginal people to give time as a demonstration of respect in the spirit of reciprocity [5,7,8,45]. Part of this respect involves clinicians approaching yarns with an understanding that there is an opportunity to learn from the Aboriginal person or family sitting in front of them [5,7,8]. The yarn is a form of reciprocity and, if the family feels comfortable to be part of this exchange, it is important to understand what a privilege this is. It is also an excellent indicator of a culturally responsive clinician. As Iningai and Bidjera knowledge holder and academic Aunty/Dr. Lorian highlighted in a two-way individual yarn:

*“How can trust someone I don’t know? If a doctor, clinician—anyone really—doesn’t understand the power of my story and the importance of listening to who I am, how can they possibly offer worthy knowledge to advise to me?”*

Importantly, it also requires a **shift in traditional clinical goals** that typically focus on outcome-based measures, to instead, the quality of the relationship as determined by the level of trust. As Jade Houghton insightfully shared about completing an assessment with an Aboriginal child:

*“I’ve learnt over time, that you have to step back and go, you know what? This kid just wants to tell me about the YouTube video he watched last night and that’s fine. And if that’s what we get through today then hey, he’s leaving happy”.*

- Family-directed yarn

When it comes to assessment and support, the knowledge review highlighted and reinforced that family-directed yarns were found to be culturally safer, more accurate and elicit far greater strengths-based, supportive and sustainable outcomes for Aboriginal families living with FASD [5,7,45]. Family-directed approaches recognise the central importance of families/kin in assessments and supports [5,7,45]. As such, this approach involves clinicians drawing on the expertise of the family/kin in collaboration to support the person living with FASD [5,7,45]. In doing so, clinicians can **gather holistic information** during an assessment, which offers an opportunity to identify and harness the strengths of Aboriginal families. In turn, these strengths can be used to **build the capacity** (i.e., knowledge, ability and confidence) **and connectedness of the whole family** to live with FASD [45].

- Applying a strengths-based wellbeing approach

All the skills and knowledge built to this point have been to equip non-Aboriginal clinicians to deliver a culturally responsive, healing-informed and trauma-aware assessment and diagnosis of FASD to Aboriginal peoples. The knowledge review found three components to achieving this. Firstly, there needs to be a focus on the strengths of individuals and their family throughout the assessment process as a way of cultivating hope, undermining stigma and building the wellbeing dimensions [2] of “purpose and control” and “dignity and respect” [5,7,8,20,40,46,51]. Secondly, the review found that it was also essential that all assessments are driven by the goal to enhance the physical, social, cultural, emotional and spiritual wellbeing of the Aboriginal person and family living with FASD, This can be achieved by drawing on and applying the dimensions of the Fabric of Aboriginal and Torres Strait Islander Wellbeing model [2]. It involves equipping clinicians with the tools and resources to strengthen the cultural, community and family supports of Aboriginal peoples undertaking an assessment and diagnosis of FASD. Finally, to practically support non-Aboriginal clinicians to facilitate a holistic (i.e., considers physical, social, cultural and emotional wellbeing), two-way yarn, where Aboriginal families feel safe and trusted to make informed decisions about things that will impact their lives, a **shared decision-making model** was identified [45,46,47,48,49,50,51,52,53,54,55,56,57]. Specifically, the “Finding Your Way” model, which was created by Aboriginal peoples for Aboriginal peoples, was used and adapted to provide non-Aboriginal clinicians the scaffolding on how to collaborate with Aboriginal families on decisions during the assessment and diagnosis of FASD [45,57].

- Advocacy

The knowledge review highlighted that, even without FASD, Aboriginal peoples are subjected to negative messages and ongoing stigma [5,7,8,41]. This stigma is often internalised if there is not a strong voice to counteract these influences across the lifespan; this is especially true in a child’s developmental years [7,9,52,53]. It was found that non-Aboriginal peoples using their inherent privilege and power to advocate for an Aboriginal person or family not only built profound trust, but was also seen to transform lives. This form of advocacy was found to change the trajectory of Aboriginal lives, potentially having a positive impact across the lifespan. As knowledge holder and Bardi academic Dr. Michael Doyle stated during a collaborative yarn:

*“For Aboriginal peoples, the escalation to child removal and justice is significantly sharper than for non-Aboriginal peoples”.*

Consequently, there are significant reforms to multiple systems of care and policy that need to be advocated for if the abovementioned findings are to be effectively implemented.. For example, until March 2023, Medicare (i.e., Australia’s publicly funded health care insurance scheme) did not include item numbers to support families in accessing assessments that can consider FASD as a possible diagnostic outcome. As a result of successful advocacy, FASD is now considered as one of the conditions under a new set of Medicare items for complex neurodevelopmental disorders.

### 3.5. What Aboriginal Communities Need to Know, Be and Do

#### 3.5.1. What Aboriginal Communities Need to Know

- Unveiling FASD

The knowledge review spoke to the “veil” (i.e., a “not knowing”), that exists around FASD in many Aboriginal communities [5,7,8,47]. This veil keeps FASD hidden and cultivates a silenceand misunderstanding or misinterpretation of particular behaviours [5,7,8]. Consistent with the general lack of FASD knowledge at a societal level, many of the yarns with Aboriginal knowledge holders highlighted the multitude of Aboriginal peoples that are not aware of the presence of FASD in their community. The first step of knowing in Aboriginal communities is the essential need for a healing-informed unveiling of FASD to occur at a local level [5,7,8]. Dr. Robyn Williams eloquently captured this in her thesis:

*“The silent story of FASD within all our families has been there for many years, waiting to be recognised and understood by society. Family narratives of FASD must be told to bring forward hope, reforms and a new era of awareness to replace what is too often described as “despair and hopelessness” by families caring for children adolescents with FASD”.*

[7] (p. 1).

Part of this unveiling requires understanding the **role of history** and how this has laid the foundations for FASD [5,7,8]. As Aunty/Dr. Lorian Hayes shared:

*“The ritual of being intoxicated due to the consumption of alcohol is not the custom of Aboriginal people. It has nothing to do with Aboriginal culture and everything to do with the hopelessness of being immersed in cycles of intergenerational boredom, learned behaviours, power struggles, crisis, vulnerability, abuse, trauma, poverty, grief and loss. We have children that wake up and drink their parents’ alcohol because they are hungry and need something to fill their bellies. Their parents did the same and their parents before that. Here, cycles of loneliness and being alone begin and our children become desperate for connection. This desperation creates gangs of lonely, hungry, isolated, traumatised children with FASD that have been starved of love and all the other basic needs. The only thing these kids are focused on is survival and that leads to a life of crime. That is not a choice, there is no freedom or self-determination in these situations”.*

It is critically important for the safety of community that unveiling FASD is facilitated collaboratively [47] and compassionately, where the community controls the **story-based yarn** [8] or **theatre-based education** [7] and sets the tone, timing, content and depth of the stories shared. Of particular importance, **humour** was found to be an effective way to increase the knowledge and awareness of FASD in a way that undermines stigma and embodies a healing-informed approach [7]. Importantly, the presence of humour facilitates another level of cultural safety and security for Aboriginal peoples. For example, Williams [7] effectively applied Aboriginal humour and language in a role play that saw attendees ”meet FASD”. Using this theatre-based, narrative therapy approach externalised FASD for the audience, allowing them to be introduced “Cheeky FASD” as a visual character and learn about FASD in way that was gentle, engaging, humorous and, ultimately, healing-informed.

#### 3.5.2. What Aboriginal Communities Need to Be

- Reframing the narrative

The knowledge review revealed that a critical ingredient to developing Aboriginal self-knowledge and behaviour (“being”) was the ability for Aboriginal peoples to compassionately explore what drives their own behaviours, while understanding, appreciating and building upon their personal strengths [7,20,40,41,50]. The term “narrative” used here refers to the ongoing individual or collective story which involves a process whereby attitudes, values, feelings, goals, skills and roles change over time and are developed to enable a fulfilling and meaningful life [7]. The knowledge review emphasised that narratives hold cultural significance and wield profound power to assert dominance or enablement depending on who is narrating the story [7]. The literature consistently highlighted that cultural identity was an important source of strength and resilience in children and adolescents with FASD [7,8,15,52]. However, the knowledge review also recognised that colonisation has given rise to deficit narratives that blame, shame and stigmatise Aboriginal peoples and therefore culture, at the collective level [5,7,8,41,52]. In turn, many Aboriginal peoples have absorbed these narratives and manifested them as **internalised colonisation** (i.e., the harmful behaviours that are done to those within an individual’s family, organisation and community as part of being consistently oppressed) [7,8,51,54]. Aboriginal peoples have sovereignty, skills and wisdom that are inherent in ancient cultural bloodlines. However, for some, this essential truth of Aboriginal beings may have been convoluted and undermined by the impacts of internalised colonisation [5,7,8]. As Dr. Robyn Williams underscored in a two-way yarn:

*“We need to take back our narratives. We lead the way in FASD research and knowledge translation, not because FASD in an “Aboriginal problem” but rather because our cultural ways are powerful, strengths-based and holistic. When the narrative lacks our Aboriginal voice and ownership, deficit discourses that focus on Aboriginal shame and blame emerge and this plays out at a grass roots level for our people. We are sovereign people and these narratives belong to us”.*

In order to reframe personal and collective narratives, the Aboriginal knowledge holders emphasised the importance of focusing on holistic Aboriginal wellbeing as a way of addressing internalised colonisation and strengthening **Aboriginal sovereignty** and **cultivating hope**, with the ultimate goal of inviting healing to each “being” [7,15,20,21,42,45,47].

#### 3.5.3. What Aboriginal Communities Need to Do

- Strengths-based pathways

The findings in the knowledge review proposed that once FASD is unveiled and the narratives and storylines reframed, Aboriginal communities would be better equipped to identify, advocate for and build strengths-based knowledge, support and referral pathways to FASD-related services that resonate with the local community. Specifically, these strengths-based pathways require **peer and personal support, National Disability Insurance Scheme (NDIS) support** and **local community-led clinical support and referral pathways.** Aboriginal strengths-based approaches to FASD may offer non-Aboriginal Australia a new lens and framework on how to view and approach disability and neurodiversity more broadly. However, the knowledge review cautioned that to do this genuinely and sustainably, the Aboriginal community-controlled sectors must lead and be appropriately resourced to do so.

- Advocacy

Most of the “doing” in local Aboriginal communities was found to be focused on advocacy and building local communities’ capacity to advocate effectively and strategically. The over-representation of Aboriginal children and adults in child protection and justice is an expression of the ongoing legacies of colonisation that unfold in the systemic inequities and racism experienced by Aboriginal peoples [5,43].

For those living with FASD, without appropriate supports, child protection and justice have become a frequent pathway, which addsfurther grief, loss and trauma to Aboriginal families and communities. As Dr. Sharynne Hamilton articulated:

*“We don’t want our kids knowing the language of the court and these systems. We want our kids knowing their languages, and the language of community and strength”.*

Advocacy may differ according to each community and the resources available, but specific systemic advocacies such as NDIS supported cultural activities at a local level, access to NDIS and FASD interpreters/liaisons were identified as potential ways to influence and secure positive and sustainable change to support Aboriginal communities living with FASD across Australia.

## 4. Discussion

The current research brings together findings gained from deeply listening to Aboriginal wisdom (strengths-based, healing-informed approaches grounded in holistic and integrated support) and Western wisdom (biomedicine and therapeutic models) to create new knowledge and practices that offer immense benefit to equity, justice, support and healing for Aboriginal families living with FASD. The FASD Indigenous Framework unfolds the changes that both non-Aboriginal clinicians and Aboriginal peoples each need to make in their respective ways of knowing, being and doing in order to facilitateaccess to healing-informed, strengths-based and culturally responsive FASD knowledge, assessments, diagnosis and support services among Aboriginal peoples. If these shifts genuinely occur, the authors expect the effects of stigma and racism will likely be minimised, and equitable access among Aboriginal peoples will be optimised.

The FASD Indigenous Framework is underpinned by peer reviewed and grey literature regarding Indigenous communities and FASD. The literature review highlighted that there is a scarcity of Aboriginal authors publishing on matters of FASD in Aboriginal communities, resulting in a distinct lack of Aboriginal peoples’ voices represented in the available literature. The current article addresses this gap by yarning with Aboriginal knowledge holders who generously shared their wisdom in the spirit of finding pathways that bring intergenerational healing and change for Aboriginal peoples across Australia. The FASD Indigenous Framework is the first of its kind in Australia and, to the authors’ knowledge, globally. It is also the first study, to the authors knowledge, to integrate a wellbeing model in the assessment and diagnosis of FASD. However, the authors recognise that while the findings of this work are unique in the field of FASD, many aspects of the FASD Indigenous Framework are well established in other Aboriginal health issues.

In a FASD context, this article uses the concept of “healing-informed” to describe how strengths-based practices that build on spiritual underpinnings of Aboriginal interconnection to culture, community, Country and kinship can be applied to bring an important layer of healing to Aboriginal peoples. Through collaborative yarning, it was revealed that it is not enough to be “trauma informed” and simply “not trigger” Aboriginal peoples. Indeed, it is time we raise the bar because true justice, reconciliation and reciprocity is embodied in the spirit of healing, not in the bare minimum practice of avoiding further harm. Similar to the Culturally Responsive Framework [35], the emphasis of healing-informed is on “doing” and overlaps with reciprocity, underpinned by Aboriginal ways of being (i.e., “ontology”). Non-Aboriginal clinicians play a critical role in creating equitable access to FASD knowledge, assessment and supports for Aboriginal peoples. Specifically, this article discusses key findings from the knowledge review. Firstly, this article will describe what non-Aboriginal clinicians can put into practice (i.e., “doing”) when they are applying a healing-informed, strengths-based Aboriginal wellbeing approach in the assessment and diagnosis of FASD. The authors acknowledge that non-Aboriginal clinicians will require training to build their knowledge, skills and confidence in applying practices such as yarning. Secondly, the discussion will suggest practical, healing-informed ways Aboriginal communities can build access to FASD knowledge, resources, services, assessments and supports. It is important to note that the FASD Indigenous Framework is a starting point, not a “solution”, to addressing FASD. The authors recognise that there are unique barriers and narratives that exist in each diverse Aboriginal community, which may limit the ability for the current findings to be applied.

### 4.1. Yarning in Practice

Yarning is a way in which non-Aboriginal clinicians can apply healing-informed communication. Healing-informed approaches enhance the cultural safety at physical, emotional and spiritual levels by the way in which they embody connection, yarning and storytelling [1,32]. These approaches serve to neutralise any power imbalances that may exist and create a culturally safe environment that is comfortable for Aboriginal peoples. However, as part of a yarn in any FASD context with Aboriginal peoples, it is essential that non-Aboriginal clinicians acknowledge history. The knowledge review emphasised the importance of non-Aboriginal clinicians knowing and genuinely acknowledging Australia’s violent history and being able to bring their cultural understandings to the forefront of a conversation with Aboriginal peoples. Research shows that failing to do so can undermine trust and inflict harm in the assessment process [43].

Storytelling is at the heart of a yarn and has played a key role in sharing knowledge and sustaining Aboriginal culture for over 60,000 years [31,32,34]. Storytelling provides a shared communication space, where plain language is used and information unfolds in a rhythm that makes sense to the natural way in which most humans understand the world [31,32]. The knowledge review consistently emphasised avoiding the use of medical or complex terminology. Using visuals, metaphors, stories and speaking plainly were found to be effective ways of translating FASD information to Aboriginal peoples, while demonstrating respect for Aboriginal cultural ways of knowing, being and doing [22,42]. Storytelling and yarning also provide a safe space for Aboriginal peoples to explore the stories of their feelings, hopes and fears and, in turn, provide opportunities for non-Aboriginal clinicians to share their stories. For many cultures, including Aboriginal peoples, storytelling is a bridge to building relationships, trust and connection, but it must be a two-way process for it to be successful. Here, begins two-way respect, connection and understanding of each other which, if nurtured with Dadirri (deep listening and quiet awareness), can lead to genuine trust.

Some clinicians trained in the Western medical system may cope with feelings of discomfort (i.e., caused by beliefs of inadequacy, incompetence and/or feelings nervousness) by overcompensating with energetic talking, which can result in a one-way speaking *at* someone rather than a two-way, *equal* exchange. This phenomenon leaves little space for the individual or family to speak, resulting in silencing the individual’s or family’s voice and diminishing their presence. This is often exacerbated by the short timeframes allocated to complete assessments due to an overwhelmed healthcare system. Wherever possible, and however small, clinicians are encouraged to change their clinical goals from a one-way, outcome-based assessment and diagnosis of FASD to the quality of a trusting, two-way connection and knowledge exchange. In doing so, clinicians could provide as much control as possible to the individual or family undertaking an assessment. In a yarn about assessment and diagnosis, it is important that the level of information exchange is controlled by the Aboriginal person or family and that they set the tone and determine what information is shared. This is fundamental to building purpose and control as part of a strengths-based approach to building Aboriginal wellbeing [2]. This is especially important considering the history and current occurrences of disempowerment that Aboriginal peoples endure even without a disability. As Jade Houghton pointed out about the Aboriginal students with FASD she supports:

*“They don’t get to be an expert in the classroom and because they don’t have those social skills, they* *don’t get to be the experts in the playground…so for them to feel like an expert, that’s pretty tight…”*

### 4.2. Yarning to Support Collaboration with Families

A second healing-informed, strengths-based and culturally responsive practice is applying the abovementioned elements of yarning to collaborate with a family, as illustrated by the Fabric of Aboriginal and Torres Strait Islander Wellbeing model and supported by previous research [3,45]. Aboriginal health and wellbeing are intrinsically tied to family and/or kinship, where belonging and connection, purpose, identity and culture are all interwoven [2]. In collaboration with the family, non-Aboriginal clinicians can support the development of a plan for the assessment process and what strategies and resources would work best to help the family. Acknowledging the natural roles and responsibilities anAboriginal person may have in their family, and building their capacity to tailor these roles and responsibilities to support the person living with FASD, offers a respectful, strengths-based approach to strengthening the connectedness of the family as a dynamic support resource. While it is important to recognise the strengths of Aboriginal families and build their capacity to access opportunities as they arise, the significant and accumulative psychosocial stress and systemic barriers experienced by the family and the impact this may have on their ability to self-advocate must be acknowledged.

Globally, there is a significant lack of FASD knowledge, support and specialised services [45]. This is profoundly exacerbated for Aboriginal peoples already experiencing exclusion, racism, discrimination, stigma, shame and guilt. This results in significant barriers to accessing the already limited FASD supports. For example, Aboriginal peoples may be reluctant to access health services because they do not want to be discriminated against [3,38]. This reluctance means Aboriginal peoples may delay attending services and only attend when they feel they have no choice (i.e., when things have become out of their control). Aboriginal people may then feel shame because they are attending a health service with significant challenges that require a high level of support. These feelings of shame can be validated by the rushed response of the health service where staff may express disapproving, impatient, or judgemental attitudes. Such negative experiences erode the family’s hope for and confidence in accessing services and supports. Yarning can provide an antidote to this by reducing the potential power imbalances and creating an understanding and supportive environment that generates cultural security for the family.

The legacies of colonisation have created a fertile ground for many Aboriginal families to be experiencing a constant state of crisis, which leads to operating in “survival mode” [45]. Consequently, these families may need material resources and support for their basic needs to be met before they have capacity to engage in practices such as reflective parenting. Further, it is important to highlight that, given Australia’s history, increasing Aboriginal caregivers’ ability to self-reflect and self-regulate will lead to a higher likelihood of processing traumatic experiences and/or coping with their own social and emotional wellbeing needs [26]. This emphasises that many Aboriginal caregivers and families will need emotional support and validation of the challenges of their lived experience [26]. Building upon the knowledge, skills and confidence of the family to live with FASD not only equips the family with increased ability to advocate and access support, but it also provides dignity, respect, purpose and control, which serve to promote hope, a critical ingredient in positive outcomes [26]. When it comes to FASD or any neurodevelopmental assessment, family-directed yarns, assessments and interventions may be culturally safer, more accurate and elicit far greater strengths-based, supportive and sustainable outcomes for Aboriginal families living with FASD [5,6,26].

### 4.3. Supporting Aboriginal Communities to Access FASD Knowledges

The FASD Indigenous Framework also shares unique insights from Aboriginal knowledge holders of the past, present and future to support the Aboriginal workforce and communities in what they need to know, be and do in order to access FASD knowledge, assessments, diagnosis and support services. First and foremost, the knowledge review highlighted that it is critical to acknowledge that alcohol use among Aboriginal peoples can only be understood within the social and historical context of colonisation, dispossession and economic exclusion [14]. While colonialism and dispossession are not the cause of all harmful alcohol use among Aboriginal peoples, observed drinking patterns are a response to this history and the current social conditions that have arisen from it [14]. Intergenerational and ongoing trauma combined with a history of exclusion from education, health and employment opportunities have created fertile grounds for ongoing cycles of poverty and feelings of helplessness [3,9,14]. In such circumstances, alcohol is often used as a soothing and coping mechanism to treat the despair experienced across the lifespan, including during pregnancy [9,14]. Understanding the histories and social structures that drive alcohol use in pregnancy is imperative to establishing and maintaining a two-way, culturally safe relationship grounded in trust and respect.

There exists a lack of awareness about FASD in general Australian society [55]; however, for Aboriginal communities specifically, there are additional historical and cultural reasons that exacerbate the barriers to accessing FASD knowledge. Historically, intergenerational cycles are compounded by the entrenched shame, guilt and stigma that have accompanied colonisation. Shame, guilt and stigma are evident across all areas of health among Aboriginal peoples, but they can be especially salient when discussing FASD and alcohol use during pregnancy [5,7]. For some Aboriginal peoples, to acknowledge the existence of FASD reinforces toxic stereotypes and invites fear, further racism and even deeper shame, guilt and stigma among Aboriginal families. Additionally, many Aboriginal families hold validated fears of having their children removed if they disclose alcohol use in pregnancy [3,38]. After all, Aboriginal children are nearly seven times more likely than non-Aboriginal children to be the subject of child protection referrals, many of which are supported by questionable evidence [3,56]. Naturally, this can have a silencing effect on Aboriginal peoples, so there remains many untold stories of alcohol use during pregnancy. The lack of records of alcohol use during pregnancy combined with a lack of awareness around FASD allow this condition to hide in the shadows of communities and perpetuate cycles of trauma, grief and loss.

### 4.4. Healing-Informed Approach to Gently “Unveil” FASD

The impact of FASD awareness within families is essential to nurturing and protecting children in communities. It is also crucial to building supportive wrap-around, healing-informed pathways to loosen the grip that colonisation continues to have on some families. The knowledge review highlighted how this must begin with arespectful, healing-informed “unveiling” of the existence of FASD in the local community. It is critically important for the safety of the community that unveiling FASD is facilitated collaboratively, where the community controls the yarn and sets the tone, timing, content and the depth of the stories shared. In the first instance, it may be inappropriate and even triggering to lead with or promote “FASD” education before community have had an opportunity to access, share and understand knowledges about alcohol use in pregnancy that resonates with their ways of knowing, being and doing. Drawing on Aunty/Dr Lorian Hayes’ grassroots approach to FASD knowledge translation, when invited into a community to yarn about alcohol and pregnancy, the Aboriginal facilitator begin by sitting and sharing their story, being ever mindful of the privilege of education they hold in this space. Using the principles of a problem-based learning approach the facilitator invites those present to yarn and deeply listens to the words spoken but more importantly, to the cultural and spiritual expressions that underpin these words [4,9].Here, insights into how a community understands and constructs knowledge can be realised. This approach allows community members to share stories and lived experiences of alcohol use and what they believe are the important issues in the community they live [4,9]. As experts on their own lives, these stories support community members to draw their own connections and conclusions, as a way of gently unveiling the inextricable link between alcohol use in pregnancy and adverse outcomes [9].

The knowledge review emphasised the critical importance of the facilitator being able to hold an intimate and intuitive healing space for unveiling FASD. To hold space is to be holistically and compassionately present to allow an environment of non-judgemental vulnerability [14]. For an Aboriginal facilitator to hold space during a yarn about FASD, and in a way that the Aboriginal community involved would determine it as safe, is dependent on several factors. It relies on the identity and reputation of the facilitator, the facilitators’ use of appropriate protocols and processes and the ability of the facilitator to be gently vulnerable and surrender any agenda they may have with regards to time, data and place [14]. As Aunty/Dr. Lorian Hayes so eloquently captured:

*“There was a lady who was very silent in the room, I sensed the rawness of her pain, I stopped the FASD training, and I encouraged everyone to get a cuppa. I went over to that lady. She hung her head, and I hung my mine, and we didn’t say much”.*

The facilitator needs to have a rich knowledge of FASD, with the ability to tailor and translate the knowledge to the diverse ways of understanding and constructing knowledge in any given Aboriginal community. The cultural authority of the facilitator (i.e., having cultural and spiritual connections, being a deeply respected knowledge holder and maintaining the highest integrity in upholding cultural protocols) was paramount to being able to hold such a space, as Aunty/Dr. Lorian emphasised:

*“It’s not about being able to play didgeridoo or paint yourself up, you need to have the cultural and knowledge authority in FASD. You need to know how a community understands knowledge—how someone communicates, interprets, and constructs this thing called “knowledge”. What does that mean to the mob? How do they construct it in their mind and body? This is going to be different for every family because each family and even those that make up that family have their own ways of knowing, being and doing. We must never forget that”.*

When facilitated well, such a space can bring profound healing to the community. The rich two-way information shared can illuminate powerful and meaningful metaphors, stories, key messages, communication pathways and innovative visual resources that will support the translation of FASD knowledge that best resonate with the local community.

### 4.5. Intuitively and Practically Applying Humour

One of the most interesting learnings offered by the knowledge review and observed by the lead author (NH) throughout the collaborative and two-way individual yarns was the way in which Aboriginal peoples intuitively and effectively use humour. Throughout the collaborative and individual yarns on FASD, there were many shared laughs, despite the often sensitive and challenging nature of the content. This showcased the incredible strength and resilience of Aboriginal peoples to be able to connect and alleviate the potential harm caused by stigma, discrimination and racism [5]. This suggests that humour is an effective healing-informed way of coping that is intuitively used by Aboriginal peoples. The use of humour in cases of Western-framed disability has been found to create a sense of belonging and connection [44], challenge stigma [45], increase the likelihood of using positive coping strategies to deal with stress [44] and alleviate tension to allow increased understanding [47]. Despite this, humour remains largely absent in creating awareness of FASD in Aboriginal communities. Humour offers an opportunity to increase access to FASD knowledge among Aboriginal communities and in a way that draws on a profound cultural strength that promotes resilience and healing. 

When it comes to translating FASD knowledge to build the capacity of the Aboriginal Health workforce and community members, Dr. Robyn Williams drew on humour and storytelling to design FASD education using a theatre-based, narrative therapy approach [5]. In a role play entitled “Yarning with Cheeky FASD”, Aboriginal language, humour and vernacular was employed and well received by both Aboriginal and non-Aboriginal peoples. “Yarning with Cheeky FASD” encouraged the participants to bring their personality and to act younger than their age. In some role plays, a mask was worn by the person acting as “Cheeky FASD”, to visually promotes the fact that FASD transcends all races and class [5]. Given that FASD training can be confronting at points, the role play provided participants with some light relief, while consolidating their learning [5]. Using this theatre-based, narrative therapy approach externalised FASD for the audience, allowing them to meet FASD as a visual character, while learning about FASD in way that was gentle, engaging and humorous [5].

### 4.6. The Powerful Strength of Sovereign Aboriginal Peoples

To draw on the sovereignty and strength of Aboriginal communities requires education and resources specific to that community. Building the capacity of Aboriginal families and communities to understand their rights and advocate for critical resources is essential to mobilising and addressing FASD. For some families and communities, advocacy will focus on local access to diagnostic and support services but for others, this may not be the case. It is important that Aboriginal peoples and their families know that if a child undertakes an assessment of any kind (e.g., FASD, Autism, ADHD), they do not have to accept the diagnosis. Every person has the right to accept or refuse a diagnosis, and it is the role of the clinician to ensure that families understand their options so that they can make an informed decision that best aligns with their beliefs, values and circumstances.

The Finding Your Way shared decision-making tool [57] can be used to build the confidence of community members to know the types of questions to ask and the kind of yarn they should be holding with a healthcare professional. If this is not the case, the knowledge review passionately encouraged Aboriginal families to seek help and support elsewhere [5]. This support may not necessarily be a health professional but someone to advocate on behalf of the family and help navigate the system. As one foster carer of a child with FASD emphasised in Williams’ thesis [7] (p. 2):

*“I would be saying jump up and down as loud as you can for the child…stay strong in your instincts, trust yourself, and ask for help, and especially with medical professionals, if at first you don’t have success try a different doctor; don’t stay with a doctor if you are not happy. Find someone who is actually going to help you and your child”.*

Accessing diagnosis of and support for FASD can be “the catalyst for change and quality of life” [7] (p. 1). Such access strengthens Aboriginal wellbeing domains of “purpose and control”, enabling Aboriginal peoples and families to have sovereignty over their narratives. 

Once FASD has been unveiled and the narratives reframed, Aboriginal communities will be better equipped to identify, advocate for and build strengths-based pathways to FASD knowledge, resources, services, assessments and supports that resonate with the local community. Past and present experiences tell us that the only way to achieve successful and equitable access to assessment and FASD diagnosis is to demand for it and continue demanding until such time as someone listens [5]. Aboriginal voices are critical to bringing about change; both the late Aunty/Dr Janet Hammill [58] and beloved Aunty/Dr Lorian Hayes [14] are testaments to this fact. It is important to highlight that the information in this article is not “new” Aboriginal knowledge; it embodies a lifetime of work and sacred knowledge held by Aunty/Dr Hammill and Aunty/Dr Lorian and reflects the pathways they relentlessly forged to bring equitable access to FASD knowledge, resources and support. By working with non-Aboriginal peoples and Western systems, they brought hope and healing to Aboriginal communities living with unrecognised FASD. In this way, these respected Elders have been drawing on Aboriginal and Western wisdom to work towards a FASD strengths-based and healing-informed practice for over 50 years. The Australian FASD Indigenous Framework is a testament to their trailblazing. All those who walk in their footsteps do so with honour and a deep sense of gratitude for the softer path.

### 4.7. Limitations

Australian Aboriginal communities are profoundly diverse, with different languages, customs and ways of knowing, being and doing. Therefore, the Cultural Advisory Group was not representative of all Australian Aboriginal communities, and the readers should be cognisant of this fact when they are reading and interpreting the results of this article. Further, a significant limitation of the current FASD Indigenous Framework is the absence of the Torres Strait Islander voices. It is anticipated that the next FASD Indigenous Framework will incorporate Torres Strait Islander voices so it has been named “Indigenous”, accordingly. It is also important to note that the artwork developed for the FASD Indigenous Framework was informed by Torres Strait Islander peoples, as well as Aboriginal peoples. Future research is encouraged to continue building on the FASD Indigenous Framework by gathering and integrating different Aboriginal communities’ perspectives and incorporating the Torres Strait Islander worldview as it relates to FASD.

## 5. Conclusions

It is critical to redress the harms from colonisation, which have laid the foundations for FASD in Aboriginal communities. While by no means a panacea to addressing FASD in every Aboriginal community, the Australian FASD Indigenous Framework presents steppingstones to guide Aboriginal and non-Aboriginal peoples’ journey together to heal these harms. Such solidarity requires changes in non-Aboriginal and Aboriginal ways of knowing, being and doing, to enable space for two-way learning, respect and trust to occur. By deeply listening to and drawing from both Aboriginal and Western wisdom, this article presents a way forward to create new knowledge and practice that offer immense benefits to the quality of assessment and support for all Australians living with FASD.

## Figures and Tables

**Table 1:** Knowledge review mapping table integrating the knowing, being and doing principles of IAHA Culturally Responsive Framework and the dimensions of the Fabric of Aboriginal and Torres Strait Islander Wellbeing model.

	Knowing and Being					Doing
	Belonging and Connection	Holistic Health	Purpose and Control	Dignity and Respect	Basic Needs	
**Culture**	Reciprocal relationships with country, family and community and the importance of culture in developing and maintaining a sense of shared experience and understanding	Multidimensional state of wellness determined and attained via the quality and balance of one’s connections to family, community and culture	Stability at home, employment and financial security, education and cultural and familial responsibilities. Family was key to a sense of stability.	How perceived and treated by others and this is associated with relationships with others, policies, services, and experiences of racism. Family provides a source of shared strength that empowers and motivates. Additionally, having non-Aboriginal systems that value and respect culture being represented positively in media.	Housing, money, access to services, education, employment, opportunities to thrive and need for justice.	Actions and considerations required to deliver a culturally responsive assessment and diagnosis of FASD
**Community**						
**Family**						

## Data Availability

The data are not available due sensitive nature of the data collected and the ability to identify the participants.

## Supplementary Materials

The following supporting information can be downloaded at: https://www.mdpi.com/article/10.3390/ijerph20065215/s1, Figure S1: Detailed FASD Indigenous Framework.
