# Supplementary figures and images for: Development of an Australian FASD Indigenous Framework: Aboriginal Healing-Informed and Strengths-Based Ways of Knowing, Being and Doing
Source: Int J Environ Res Public Health. 2023 Mar 22;20(6):5215. doi: 10.3390/ijerph20065215 (PMC10049125; doi:10.3390/ijerph20065215)

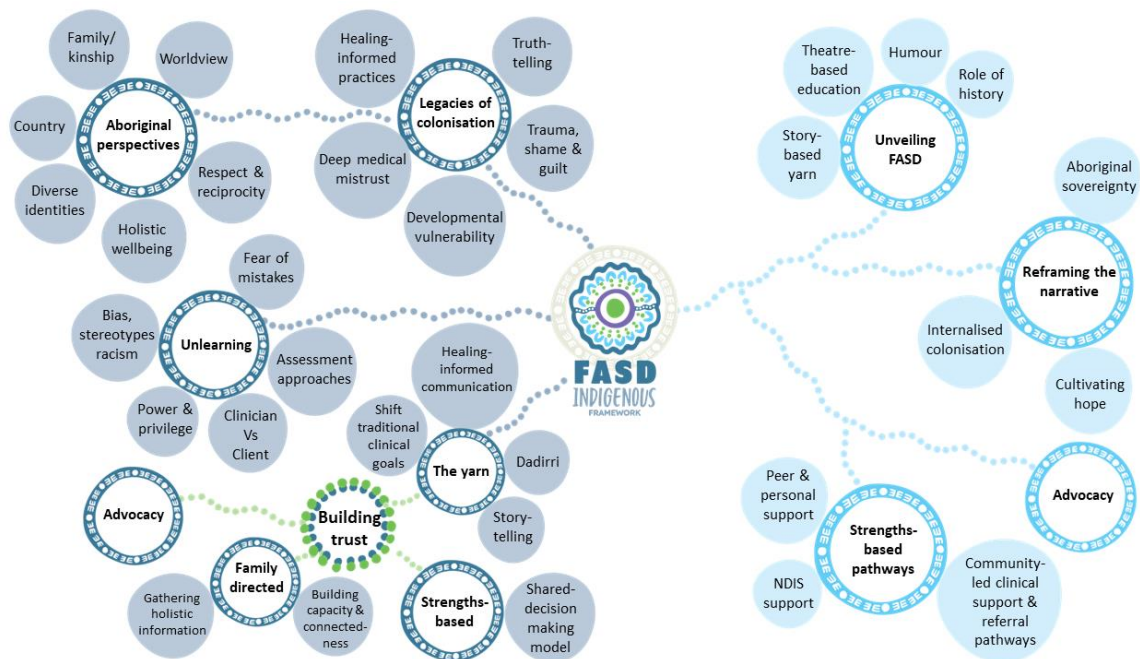

Figure S1. Detailed FASD Indigenous Framework.

Supplement: Supplementary file 1 [file ijerph-20-05215-s001.zip › ijerph-2220702-supplementary.pdf]
